# Encouraging responsible reporting practices in the *Instructions* to *Authors* of neuroscience and physiology journals: There is room to improve

**DOI:** 10.1371/journal.pone.0283753

**Published:** 2023-03-30

**Authors:** Joanna Diong, Elizabeth Bye, Zoë Djajadikarta, Annie A. Butler, Simon C. Gandevia, Martin E. Héroux

**Affiliations:** 1 School of Medical Sciences, Faculty of Medicine and Health, The University of Sydney, Sydney, NSW, Australia; 2 Neuroscience Research Australia (NeuRA), Sydney, NSW, Australia; 3 School of Medical Sciences, University of New South Wales, Sydney, NSW, Australia; 4 Clinical School, University of New South Wales, Sydney, NSW, Australia; Universitat Oberta de Catalunya, SPAIN

## Abstract

Journals can substantially influence the quality of research reports by including responsible reporting practices in their *Instructions to Authors*. We assessed the extent to which 100 journals in neuroscience and physiology required authors to report methods and results in a rigorous and transparent way. For each journal, *Instructions to Authors* and any referenced reporting guideline or checklist were downloaded from journal websites. Twenty-two questions were developed to assess how journal *Instructions to Authors* address fundamental aspects of rigor and transparency in five key reporting areas. Journal *Instructions to Authors* and all referenced external guidelines and checklists were audited against these 22 questions. Of the full sample of 100 *Instructions to Authors*, 34 did not reference any external reporting guideline or checklist. Reporting whether clinical trial protocols were pre-registered was required by 49 journals and encouraged by 7 others. Making data publicly available was encouraged by 64 journals; making (processing or statistical) code publicly available was encouraged by ∼30 of the journals. Other responsible reporting practices were mentioned by less than 20 of the journals. Journals can improve the quality of research reports by mandating, or at least encouraging, the responsible reporting practices highlighted here.

## Introduction

To gain knowledge, truthful and accurate reporting of scientific discovery is needed. At a minimum, research should be designed and conducted rigorously, and reported with sufficient detail so that findings can be independently verified. Transparent reporting of methods and results goes a long way towards scientific trustworthiness.

Unfortunately, the transparency and quality of research reporting are often sub-standard. For example, despite widespread adoption of the Consolidated Standards of Reporting Trials (CONSORT) checklist, it is adhered to by only 65% of randomised controlled trials published in rehabilitation journals [1]. In line with this, 20–37% of rehabilitation trials do not adequately report how they randomised participants or calculated their sample sizes [2]. The reporting of pre-clinical research is also problematic and includes the inadequate reporting of statistical tests (45%), software (31%) and sample size (44%) [3]. Our own team has highlighted issues related to research reporting in physiology and neuroscience. For example, an audit of papers published by researchers from a neuroscience research institute found that 30% of papers included measures of variability (or confidence) that were not defined [4]. This issue was also present in 15% of papers in two preeminent physiology and pharmacology journals [5], as well as 13% of papers in a leading neurophysiology journal [6]. The standard error of the mean is often misused to summarise data variability [7], a practice that was noted in 32% of papers from a neuroscience research institute [4], 79% of papers published in a physiology journal and a pharmacology journal [5], and 65% of figures published in a leading neurophysiology journal [6]. Other common issues that we highlighted were the failure to define the threshold value used for statistical tests (38%) [4], the failure to report exact p-values (40–58%) [4, 6], and ‘spin’ (57–59%), the misleading interpretation of statistically non-significant findings (e.g. p-values between 0.05–0.1) [5, 6].

One strategy to improve the transparency and quality of research reports has been to introduce reporting guidelines and checklists. Developed by consensus working groups, these guidelines provide a minimum list of criteria that ensures scientific papers are rigorously and transparently reported. The EQUATOR Network (Enhancing the QUAlity and Transparency Of health Research; www.equator-network.org), an international initiative that seeks to improve the reliability and value of published health and medical research, hosts and disseminates reporting guidelines for many common study designs. Well-known reporting guidelines include CONSORT [8] for randomised trials and ARRIVE [9] for animal pre-clinical studies. Additionally, some publishers, professional societies and journal editorial boards use in-house reporting guidelines or checklists to improve research reporting. Examples of these include checklists by the Nature Publishing Group [10] and the American Physiological Society [11], and editorial recommendations by the Journal of Physiology [12–14].

Unfortunately, reporting guidelines and checklists have not solved the problem of poor scientific reporting. Reporting guidelines are often specific to a given study design, and they typically do not address fundamental aspects of scientific reporting, such as those highlighted above. Moreover, they are not necessarily used or adhered to by authors [1, 15–18]. While editorial advice encouraging improved reporting is well intentioned, it is also generally ignored by authors [5].

Thus, the responsibility of ensuring transparent and high-quality research reporting rests squarely on the shoulders of journal *Instructions to Authors*. Unfortunately, these issues are not necessarily addressed in *Instructions to Authors*. For example, a large audit that spanned dozens of scientific disciplines found that most *Instructions to Authors* fail to address key aspects of responsible research reporting [19]. Among *Instructions to Authors* of health and life science journals, only 32–35% addressed data sharing, 22–38% addressed study registration and only 6–16% addressed the most minimal of statistical reporting requirements.

Based on the available evidence, journal *Instructions to Authors* appear to incorporate few requirements and recommendations to improve research reporting. It is not known whether journals in neuroscience and physiology fare any better. Therefore, the aim of the present study was to assess the extent to which journals in neuroscience and physiology address responsible reporting practices. Specifically, we examined the rigor and transparency that journals require of their authors when they report their methods and results.

## Materials and methods

Eligible journals were identified using the Journal Citation Reports database via Web of Science, at the University of Sydney Library. Journal titles in the Journal Citation Reports categories of ‘Neuroscience’ and ‘Physiology’ that were listed in 2019 with an impact factor of at least 1.00 were identified. This allowed us to assess *Instructions to Authors* of journals that have been in publication for several years and have some influence in these fields. Moreover, journals that published only reviews were not considered as we wanted the sample of *Instructions to Authors* to reflect instructions by journals that focused on original research.

In total, 320 eligible journal titles across both neuroscience and physiology were identified, of which 100 were retained for analysis. Eligible journal titles were first independently and purposefully sampled by senior investigators (AB, JD, MH, SG), based on their experience, to reflect the breadth of research and readership in these fields (n = 158). Of these, titles selected by at least 2 investigators were shortlisted (n = 39), and an additional 61 journal titles were randomly sampled.

Journal impact factors and the number of citable items for these 100 journal titles were obtained. Next, journal titles were allocated to each of the 6 investigators. Investigators downloaded the *Instructions to Authors* over a 2-week period (22 Apr to 7 May 2021). Either the text of the *Instructions to Authors* was copied to a Microsoft Word file, or the PDF of the *Instructions to Authors* was retrieved. All referenced reporting guidelines or checklists were noted for all 100 journals. Examples of referenced reporting guidelines that were downloaded by investigators included those indexed on the EQUATOR Network (www.equator-network.org), checklists endorsed by professional associations or required by editorial policy, and recommendations from editorial series. However, external reporting guidelines and checklists were not retrieved if *Instructions to Authors* did not specifically encourage or require their use. For each journal, document retrieval was checked by a second investigator to ensure completeness.

### Question development and pilot testing

Twenty-two questions and scoring criteria were developed to assess how strongly journal *Instructions to Authors* addressed responsible reporting practices. The questions were developed using the Statistical Analyses and Methods in the Published Literature (SAMPL) guidelines [20] and questions from our previous audits of journal reporting practices [4–6]. They focused on fundamental aspects of the reporting of research methods (Q1–4), statistical methods (Q5–8), and results in text (Q9–17), the plotting of results in figures (Q18–19), and making data and code publicly available (Q20–22).

The wording of the questions is shown in Table 1. In a pilot test, a random sample of 8 journal *Instructions to Authors* was used to assess agreement between investigators and refine the wording of the questions. The text of journal *Instructions to Authors* was assessed to determine if questions were satisfied using these scores: *Not mentioned*, *Encouraged*, *Required*.

Questions were scored as *Not mentioned* if the specific reporting practice was not encouraged or required in the *Instructions to Authors*. Questions were scored as *Encouraged* if *Instructions to Authors* used words such as ‘encourage’, ‘recommend’, ‘suggest’, and ‘advise’. Questions were scored as *Required* if *Instructions to Authors* used words such as ‘require’, ‘mandate’, ‘should’, and ‘must’. Scores that differed between investigators were discussed to achieve agreement and consistency. The wording of the questions was refined to improve consistency between investigators.

**Table 1:** Questions to assess how strongly journal *Instructions to Authors* addressed responsible reporting practices, and variable names (in *italics*) used in Fig 1.

**When reporting research methods, do the Journal Instructions require authors to:**	
Q1. report whether the study protocol was pre-registered, for clinical trials?	*prereg trial*
Q2. report whether the study protocol was pre-registered or registered, for all other study designs?	*prereg other*
Q3. report all statistical tests performed?	*stat tests*
Q4. report all outcomes?	*outcomes*
**When reporting statistical methods, do the Journal Instructions require authors to:**	
Q5. report, for frequentist analysis, the alpha threshold (e.g. 0.05) that defines statistical significance?	*alpha*
Q6. report criteria used to exclude data?	*exclude*
Q7. report how any missing data were treated in the analyses?	*missing*
Q8. report, for Bayesian analysis, the prior probabilities?	*prior*
**When reporting results in text, do the Journal Instructions require authors to:**	
Q9. define all written measures that describe data variability?	*var*
Q10. describe data using mean and SD, where appropriate?	*mean*
Q11. describe data using median and IQR, range, or both, where appropriate?	*median*
Q12. NOT use the SE or CI (e.g. 95% CI) to describe data?	*no SEM*
Q13. report the P value for all tests of statistical significance?	*p val*
Q14. report exact P values (e.g. p = 0.021 or p <0.001)?	*exact p*
Q15. report a measure of precision (e.g. 95% CI) for effect sizes?	*precision*
Q16. NOT interpret P values above the alpha threshold as trends towards statistical significance or as statistically significant?	*trend*
Q17. report, for Bayesian analysis, the posterior distribution with a measure of central tendency and a credibility interval?	*posterior*
**When plotting results in figures, do the Journal Instructions require authors to:**	
Q18. define all plotted measures that summarise data variability or estimates of effect size?	*var*
Q19. plot the individual participant/ specimen data in all figures that summarise data variability or estimates of effect size?	*raw data*
**To encourage transparent reporting, do the Journal Instructions require authors to:**	
Q20. make (de-identified) data used in the statistical analysis publicly available?	*pub data*
Q21. make data processing code or spreadsheets publicly available?	*pub code*
Q22. make statistical analysis code or spreadsheets publicly available?	*pub stat*

### Audit of *Instructions to Authors*

Each investigator was randomly allocated 15 to 20 *Instructions to Authors* to audit. If they encountered a statement that was unclear, or they were unsure how to score it (i.e. encourage or require), the team discussed the issue and a consensus decision was reached. *Instructions to Authors* were also assessed for inconsistencies: for example, if they directed authors to report results as mean (SD), but included an example of a figure that reports mean (SE).

### Audit of external reporting guidelines and checklists

Some *Instructions to Authors* direct authors to comply with external reporting guidelines and checklists, some of which partially address our 22 questions. This directive is important as it is a second mechanism, in addition to directives in journal *Instructions to Authors*, that can help improve reporting practices. Therefore, we also audited the 36 external reporting guidelines and checklists referenced by journal *Instructions to Authors*. Some external reporting guidelines were available in a main form, and in various extensions. For example, the PRISMA guideline that is used to report systematic reviews has an extension, the PRISMA-P guideline, that is used to report protocols of systematic reviews. To simplify this part of the audit, only the main form of guidelines were audited. Given that reporting guidelines (and some checklists) are not necessarily applicable to all papers published in a given journal, their results are reported separately.

### Data analysis

Results were tallied for each of the 22 audit questions and reported for the full sample of *Instructions to Authors*, as well as the subset that did not reference any external guidelines or checklists. We also report which external guidelines and checklists were referenced by individual journal *Instructions to Authors*, and how each external guideline and checklist scored when audited against the 22 questions.

Descriptive data are reported. All data processing and analysis were performed using Python (v3.9). The study protocol was pre-registered on the Open Science Foundation (https://osf.io/wtkzy/). Raw data, computer analysis code and results are available from the project repository (https://github.com/joannadiong/Diong_et_al_2023_PLOSOne).

## Results

The full sample consisted of 100 *Instructions to Authors* from journals in neuroscience and physiology. The median (IQR) impact factor was 3.04 (2.21 to 4.57), and the number of citable items was 7,157 (3,723 to 21,989). Of the 100 *Instructions to Authors*, 34 did not reference any external reporting guideline or checklist.

**Fig 1:**
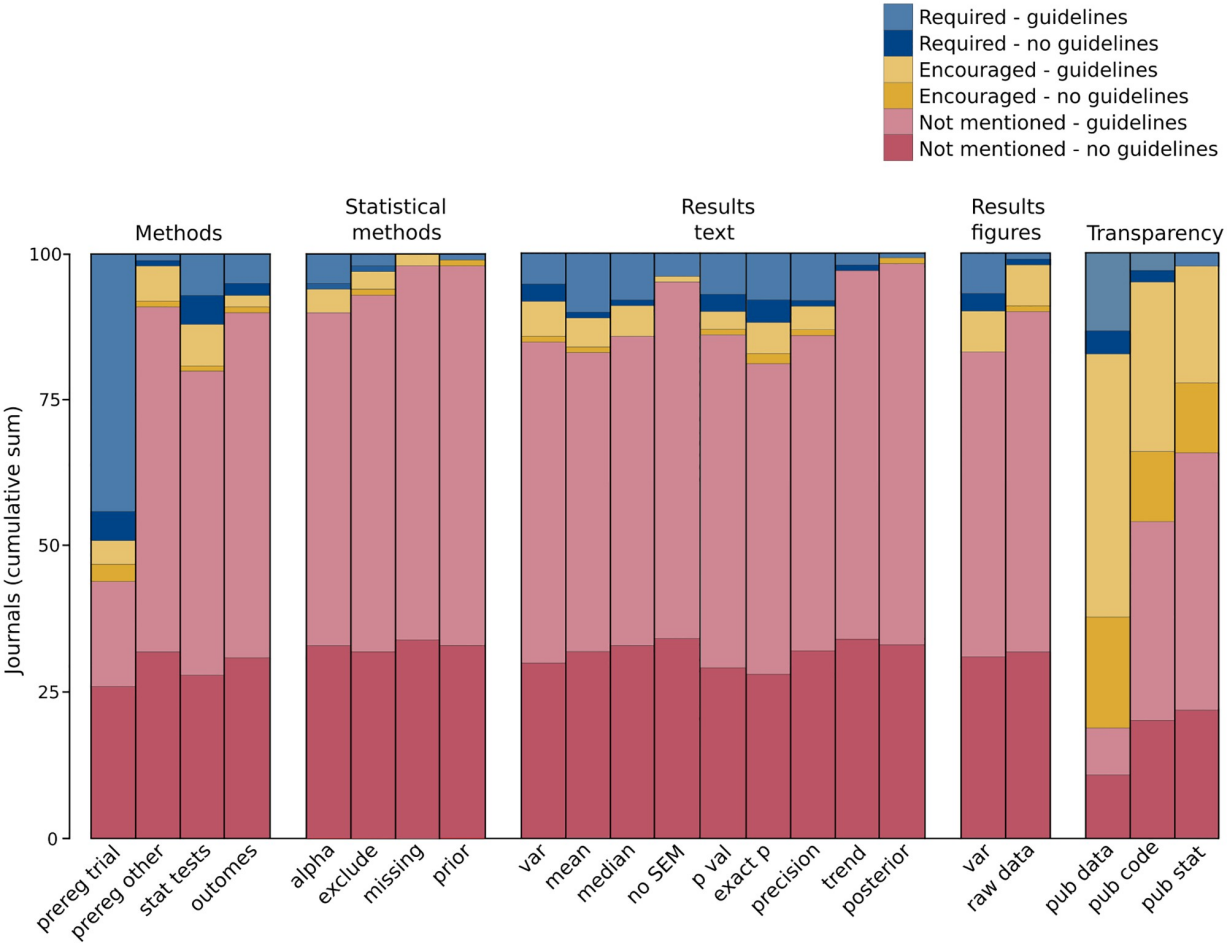
Audit results. Number of journals that addressed responsible practices in reporting research. Scores of *Required*, *Encouraged* and *Not mentioned* are presented stratified by whether journals referenced external guidelines and checklists (*- guidelines*; lighter colours) or did not reference external guidelines and checklists (*- no guidelines*, darker colours). Refer to Table 1 for wording of questions and variable names in this figure.

Audit results are presented in Fig 1. Scores of *Not mentioned*, *Encouraged* and *Required* are presented stratified by whether journal *Instructions to Authors* did or did not reference external guidelines and checklists. The data used to generate this figure are provided separately (S1 File).

When reporting research methods, the most commonly addressed reporting practice in *Instructions to Authors* was to report whether the study protocol was pre-registered, for clinical trials (required: 49/100, encouraged: 7/100). This practice was addressed much more widely than the second most commonly addressed practice to report all statistical tests performed (required: 12/100, encouraged: 8/100).

Few practices to report statistical methods were commonly addressed. The least commonly addressed practice was to report how any missing data were treated in the analyses (encouraged: 2/100). The most commonly addressed practice was to report, for frequentist analysis, the alpha threshold that defines statistical significance (required: 6/100, encouraged: 4/100).

Also, few practices to report results in text and figures were commonly addressed. The least commonly addressed practice was to report, for Bayesian analysis, the posterior distribution with a measure of central tendency and a credibility interval in text (required: 1/100, encouraged: 1/100). The most commonly addressed practices were to report exact P values in text (required: 12/100, encouraged: 7/100), and to define all plotted measures that summarise data variability or estimates of effect size in figures (required: 10/100, encouraged: 7/100).

Reporting practices to encourage transparent reporting were addressed the most widely. The most commonly addressed practice was to make (de-identified) data used in the statistical analysis publicly available (required: 17/100, encouraged: 64/100). However, practices to make data processing code or spreadsheets publicly available (required: 5/100, encouraged: 41/100), and to make statistical analysis code or spreadsheets publicly available (required: 2/100, encouraged: 32/100), in comparison to practices in other sections, were also widely addressed.

The 36 external guidelines and checklists referenced by the journal *Instructions to Authors* are listed in S1 Table, and their audit results are summarised in Table 2 and provided in S2 Table. There was substantial variation in the rigor and transparency required by external reporting guidelines and checklists. The most commonly addressed practices were to report all statistical tests performed (encouraged: 6/36; required: 11/36), and report a measure of precision (e.g. 95%CI) for effect sizes (encouraged: 3/36; required: 14/36). However, most aspects of reporting rigor and transparency were not addressed.

**Table 2:** Summary results across the 36 external guidelines and checklists referenced by journal *Instruction to Authors*. Values represent the number of external guidelines and checklists that required, encouraged or did not mention audit question items. Question names refer to those defined in Table 1. Audit results for each external guideline and checklist are provided in S2 Table.

Questions	Required	Encouraged	Not mentioned
Q1. *prereg trial*	3	2	31
Q2. *prereg other*	2	3	31
Q3. *stat tests*	11	6	19
Q4. *outcomes*	6	5	25
Q5. *alpha*	2	1	33
Q6. *exclude*	8	0	28
Q7. *missing*	8	6	22
Q8. *prior*	1	6	35
Q9. *var*	3	2	31
Q10. *mean*	3	4	29
Q11. *median*	3	0	33
Q12. *no SEM*	1	2	33
Q13. *p val*	1	1	34
Q14. *exact p*	2	2	32
Q15. *precision*	14	3	19
Q16. *trend*	0	0	36
Q17. *posterior*	1	0	35
Q18. *var*	0	2	34
Q19. *raw data*	0	3	34
Q20. *pub data*	2	5	29
Q21. *pub code*	2	4	30
Q22. *pub stat*	2	4	30

## Discussion

We examined whether journals in neuroscience and physiology require, or at the very least encourage, authors to adopt responsible reporting practices. In particular, we assessed fundamental aspects of scientific reporting that are not typically addressed by reporting guidelines and checklists. We found that most journal *Instructions to Authors* do not adequately address these practices, with the exception that approximately half of the journals asked authors to make data and code publicly available, and to indicate whether clinical trial protocols were registered.

Our findings are broadly consistent with findings from other audits of journal *Instructions to Authors* in emergency medicine [21], nutrition and dietetics [22], and other diverse scientific disciplines [19]. External reporting guidelines and checklists are widely encouraged or required in nutrition and dietetics (91%), moderately in neuroscience and physiology (66%; current study) and emergency medicine (56%), but to a lesser extent in health (36%) and life sciences (19%). Registration of clinical trial protocols is often encouraged or required in neuroscience and physiology (56%), emergency medicine (44%), and nutrition and dietetics (82%). Transparent reporting of statistical methods and results was encouraged or required to a similar extent in neuroscience and physiology (2 to 19%) and in health (16%) and life sciences (6%). However, data sharing was encouraged or required more often in neuroscience and physiology (81%) and in nutrition and dietetics (76%), than in health (32%) and life sciences (35%).

Why are responsible research practices not implemented systematically or more widely by journals? It is likely that editors are aware of these fundamental aspects of rigorous and transparent reporting. However, they may incorrectly assume that researchers are also aware of them, and that authors (not journals) are responsible for implementing them. Editors may also be reluctant to implement responsible reporting practices, just as they are reluctant to address scientific misconduct or rectify publication errors [23–25]. Also, editors may not agree on the importance of each reporting practice, or which ones should be implemented in their *Instructions to Authors*.

In other cases, editors may be overly enthusiastic and inadvertently overwhelm authors with too many instructions. This may occur when *Instructions to Authors* are administered by a succession of editors, each adding more and more instructions, often through circuitous hyperlinks and nested hyperlinks. Without proper organisation, *Instructions to Authors* can become so onerous and confusing that authors have trouble locating and extracting key information, and therefore are unable to comply with requirements. This becomes a barrier for authors to implement responsible reporting practices. Thankfully, there are examples of journals that have managed to find a balance. For example, the *Instructions to Authors* for the journal *Spinal Cord*, which are available as a simple downloadable PDF, are streamlined, well organised, and address key responsible reporting practices.

Even if responsible reporting practices are encouraged by journals, researchers may be unwilling to fundamentally change. Our previous audit showed that editorial recommendations alone are not sufficient for reform [5]. In another example, a journal went to great lengths to have authors report confidence intervals of estimates rather that uninformative p values [26]. While sole reliance on p values decreased from 63% to 5%, and the reporting of confidence intervals increased from 10% to 54%, compliance was superficial: when interpreting findings, few authors discussed the precision of their estimates as indicated by the width of the confidence intervals. That is, authors reported estimates of effects and precision simply to comply with journal policy.

Our study is not without limitations. We purposefully sampled journal titles to identify key journals in physiology and neuroscience. This was done based on the experience and familiarity of senior investigators in these fields (∼85 years of combined experience), and random sampling of journal titles with impact factors of at least 1.00. However, we may have missed influential journals, and the extent to which their *Instructions to Authors* address responsible reporting practices would not be reflected in our findings. In addition, our findings are based on the text in journal *Instructions to Authors* and reporting guidelines and checklists downloaded over a 2-week period in early 2021. If journals had made improvements to their *Instructions to Authors* to address responsible reporting practices *after* this date, these changes would not be reflected in our findings.

Even so, what can be done to improve responsible reporting practices? One strategy would be to incorporate additional items into existing guidelines and checklists. Unfortunately, this seems unlikely to gain broad support. Another strategy would be to devise a new reporting guideline. However, to improve the quality of scientific reporting, this guideline would need to be adopted and enforced broadly. Given the large number of guidelines already in existence, and the fact that relevant guidelines are not necessarily used or adhered to [1, 15–18], it is unclear whether such an approach would improve the *status quo*.

Another suggestion would be for editors to shortlist a set of key reporting practices that are uncontroversial and easy to implement (e.g. reporting whether the study protocol was registered, reporting the alpha threshold, reporting exact p values, plotting all individual data in figures, prohibiting ‘spin’) and mandate these practices on manuscript submission. Then, on first submission, manuscripts could be assessed by junior editors for compliance with these practices.

Why junior editors? Reviewers’ time is precious and in high demand. Moreover, they review papers from different journals, each with different reporting requirements. Thus, reviewers are unlikely to accept the responsibility of ensuring authors comply with journal reporting requirements. Editors are in a better position to assess and mandate journal-specific reporting requirements, and there is evidence that editorial interventions can, in some cases, lead to positive changes in reporting practices [26, 27]. However, editors’ time is also precious and, at least for some journals, they are not required to carefully read the manuscripts they handle. Finally, journal publication staff may not possess the knowledge and expertise needed to assess compliance with reporting practices. Thus, junior researchers—PhD students, post-doctoral researchers and junior faculty members—are ideally suited to help at this compliance stage, which would provide valuable editorial experience and professional connections.

It is important to acknowledge that many journals are owned by large publishing companies that may implement a standardised set of *Instructions to Authors* across these journals. Thus, editors may not be able to influence the content of the *Instructions to Authors*. In these circumstances, editors and publishers could work to improve reporting practices by educating readers on the importance of these fundamental aspects of scientific reporting, by using editorials or special interest series to generate discussion.

More broadly, editors who are part of a journal collaborative could work together to improve reporting practices. For example, editors of 28 major rehabilitation and disability journals joined forces to take a more aggressive stance on the use of reporting guidelines [28]. They simultaneously published an editorial of the collaborative agreement, which requires authors to use relevant EQUATOR reporting guidelines when preparing manuscripts for submission, and asks reviewers to use reporting guidelines when reviewing manuscripts [29]. Nevertheless, mandating responsible reporting practices does not imply authors will necessarily appreciate their importance. A step in the right direction would be for senior researchers to adopt and promote responsible reporting practices in their own work, within their institutions, and their societies and journals.

## Conclusion

Journals can substantially influence the quality of research reports by preventing poor reporting practices. A simple approach to achieve this would be for journals to mandate a set of key reporting practices that are most easily addressed. In time, this will hopefully translate to better reporting standards in neuroscience and physiology.

## Supporting information

S1 File Scores of journal *Instructions to Authors* when audited against the 22 questions. (TXT)

S1 Table External reporting guidelines and checklists referenced by journal *Instructions to Authors*. (CSV)

S2 Table Scores of external guidelines and checklists when audited against the 22 questions. (XLSX)
